# Medicine

**Published:** 1899-09

**Authors:** George Parker


### Progress of the Medical Sciences.

### MEDICINE.

Common as **malaria** is in Bristol in returned sailors and soldiers, nothing is more remarkable than the disappearance of the home-grown disease from England in the last fifty years, and it is to be hoped that our neighbours in the marsh districts will exert themselves to discover whether those mosquito gnats have actually vanished from this country which have been shown to transmit it. Stephens found two species of anopheles here in 1825, and an investigation has been set on foot to determine whether they, like the malaria, have ceased to multiply on British soil.

Thin [1] points out that one great objection to the **mosquito theory** of malaria fell to the ground when it was shown that only certain species conveyed it, for both the fever districts and the healthy ones in Sicily swarm with mosquitos. Grassi [2] found that in Italy Culex pipiens swarmed in places where there was no human malaria; but where this disease existed the anopheles, or else the Culex penicillaris or Culex malariæ abound. Ross had already shown that C. pipiens was the form which produced malaria in sparrows, and had traced the development of the avian parasites in its body. The Italians did the same with the human plasmode in anopheles, and last autumn they produced malaria in a healthy man, who volunteered for the experiment, by shutting him up with these mosquitos which had been brought from an infected district. It has been shown that the young ones when hatched in a laboratory cannot convey the disease; but if they bite an affected person, and are kept some days in a warm temperature, they will then produce in a previously healthy person the exact type of malaria from which the first man was suffering. Thus experimental proof has been given of two points: the first, that the parasite can pass from the human body to a second host in certain mosquitos; and secondly, that mosquitos can produce the disease in men. Koch notices that in the central parts of Rome malaria does not appear, although the water comes from malarial districts; and Bignami and Grassi bring an argument against the theory of air-borne infection, showing that the plasmodes cannot bear the least desiccation.[3] Against the actual facts of the Italians, Lawrie's theoretical objections have little weight. So late as last December we find him asserting that the Laveran bodies are merely degenerate blood cells.[4] Thayer and Hewetson found them in every one of 616 cases of malaria except two or three convalescents.[5] Still, Ewing[6] acknowledges that occasionally they cannot be met with in a typical case of æstivo-autumnal fever; but even then a search in the spleen will sometimes reveal them, just as in similar cases of typhoid.

According to Ross,[7] the **life history of the plasmode** commences with its existence as an amœbula in a red cell which develops either asexually by spores for several generations in the same host; or, by gametocytes, such as the crescents, which, taken up by mosquitos, show male and female forms.

[1] *Brit. M. J.*, 1899, ii. 349.
[2] Quoted in Editorial, *Brit. M. J.*, 1898, ii. 1767. [3] *Ibid.*
[4] *Lancet*, 1898, ii. 1468
[5] *Johns Hopkins Hosp. Rep.*, 1895, v. 5. [6] *N. York M. J.*, 1899, lxix. 149.
[7] *Nature*, 1899 lx. 323.

Filaments from the male forms attack the female ones. The fertilised body is termed a zygote, which grows in the stomach-wall of the mosquito, and finally produces the flagellulæ or zygotoblasts. These pass into the salivary duct and stylet of the mosquito, and are introduced into a new host. If it can be shown that we can extirpate the malaria-bearing species of mosquito in a given locality, and that they are only a few out of many, the propagation of the parasites and of the disease may be stopped. It is claimed that their breeding places are comparatively few, and that the larvæ are killed by a few drops of paraffin thrown into the pool where they live. However, the subject is yet in its infancy, and one in which Bristol men may find an ample field for study.

**Gastric ulcer** is clinically accompanied in most cases by hyperchlorhydria, which, if not the cause, at any rate has great influence in prolonging the condition. Hence the value of **treatment directed against the excess of acid,** such as feeding entirely by the rectum and the use of the fixed alkalies, as magnesia and carbonate of bismuth, to neutralise the secretion without stimulating it as soda does. Milk, too, is valuable from its rapid combination with the free acid. Thus Frèmont [1] gives an ounce of warm milk with bismuth and magnesia every half-hour for twenty hours to be increased if the pain is not relieved. Olivetti [2] finds that the plan of giving massive doses of bismuth, 2½ to 5 drachms, every morning on an empty stomach, for which so much has been claimed, produces little change in the motility or secretions of the organ ; while Soupault declares that sodium chlorate in divided doses of two drachms daily, given when the stomach is empty, affords great relief in ulcer or hyperchlorhydria.

Tripier,[3] to cope with **hemorrhage,** employs rectal injections of hot water at a temperature of about 120° every few hours, and allows no food to be given by the mouth. A form of ulceration, the minute erosion of gastric arteries, originally described by Murchison, is of interest from the absence of the usual dyspeptic symptoms beforehand. Dieulafoy and Lindsay Steven [4] have each pointed out that it may be the initial form of the ordinary round ulcer. The condition however has been rarely seen, and similar cases have been referred to vaso-motor disturbance. There is merely a small superficial abrasion with an opening into a vessel, easily overlooked. The hemorrhage may be extremely profuse; one of Steven's patients lost ninety-six ounces, and in another gastrotomy was unsuccessful from the difficulty of finding the bleeding point. The diagnosis would have to be made from ordinary ulcer, varix of the stomach or œsophagus, cancer, hyperæmia in heart disease, and duodenal ulcer.

[1] *Am. J. M. Sc.*, 1898, cxvi. 610. [2] *Brit. M. J.*, 1898, ii. Epitome, p. 103. [3] *Brit. M. J.*, 1899, i. Epitome, p. 12. [4] *Glasgow M. J.*, 1899, li. 5.

**Renal tuberculosis** has been largely relegated to the care of the surgeon, partly as a local affection which should be removed as early as possible lest the bladder and general system should be affected, and partly from the little success which has attended medical treatment of tubercular diseases in the past. It is an interesting question, as our treatment of phthisis becomes more and more perfect, how far this view must be altered. In other words, can we expect such local affections, at least in their earliest stages, to disappear under the increased resisting power of the tissues brought about by fresh air, increased nourishment, climatic and other aids? How many instances of spontaneous cure and of recovery, more or less complete, under improved conditions of general health are on record? Enough to show that as great results may be hoped for with the improved methods as in phthisis.

The kidney is attacked either above from the blood-stream or below from the bladder, prostate, and testes, and practically never by direct invasion from a neighbouring tubercular focus such as a suprarenal one. In the **descending type** the first lesions are seen in the cortex or as minute tubercles under the mucous membrane of the pelvis,[1] and the disease may take the miliary or caseous form, or produce a pyelo-nephritis. The bacilli have been demonstrated in the glomeruli before any pathological changes were observable, and the possibility of their frequent passage in tuberculous patients through the kidney without injuring it is to be considered. Tilden Brown[2] seems to think this may sometimes be the case, but he does not give any instances where bacilli have been found in the urine and the kidneys after death proved to be normal. On the other hand, he points out that in descending tuberculosis the disease in the kidney may be far advanced before any symptoms appear.

The absence of symptoms in the descending form previous to an eruption into the calices is in marked contrast to the violent pain and dysuria which occur in early stages when the infection **ascends** from the bladder.

If, then, we have often no means of diagnosing an early attack, it may be asked whether we may expect the disease in any definite **percentage** of tuberculous patients. Chambers found it occurred in 18 per cent, Tilden Brown in 34, Collinet 5.5, and the Prague statistics give 5.6. But even if these figures were trustworthy they would be of little use, because the disease is so often the first or the only manifestation of tuberculosis, and we do not know the relative frequency of these primary and secondary cases. In doubtful instances some importance clearly attaches to a tubercular history or the presence of disease in the lungs, epididymis, bladder, peritoneum, or intestines; but it has been experimentally produced by

[1] Bryson, in *A System of Genito-Urinary Diseases* [*etc.*]. Edited by Prince A. Morrow, vol. i. part ii., 1893, p. 837.

[2] *N. York M. J.*, 1897, lxv. 377.

injection of bacilli into the aorta by Borrell, and the mode of entrance into the system may be undiscoverable. We can only say that renal tuberculosis has been found in from 1 to 2 per cent. of all necropsies in 5,000 cases by Morris and Carmargo; it is more common in men than in women, and rare in infancy. In about half the cases both kidneys are affected, and at an early stage the prostate will show one or two hard nodules in almost every case, according to Bryson,[1] even when the disease began in the kidney. The opinion is generally held now that the descending form is the prevalent one, though Rokitansky and many others took the opposite view. All authorities however agree that the prostate rarely escapes, and emphasise the importance of its examination. Hamill,[2] who collected fifty-five cases in children under 14, finds that in them the disease is almost always of the descending type, genital tuberculosis as a starting point being certainly rare at this age.

When **symptoms** do occur, they may include paroxysmal or continued pain in any part of the urinary tract, hæmaturia with acid urine, the presence of a tumour, the signs of bladder irritation, and tubercle bacilli. Brown remarks, as important in an early stage, the curious pallor of the face, a slight evening rise of temperature, and an increase in the water excreted. The last is probably compensatory to the destruction of part of the kidney, as shown in Rose Bradford's experiments. Casper[3] notes that there is sometimes an entire absence of pyrexia throughout the disease, and quotes four such cases. Generally in later stages, and with septic kidneys, marked hectic with irregular chills occur, with emaciation and weakness. Tube casts are usually absent; albumin derived from the blood and pus cells is found when actual erosions have taken place into the urinary tract, but it is not in excess of that due to the pus or hæmoglobin present. Occasionally it is found with casts, but without blood, at an early stage from a secondary nephritis, but generally the remaining tubules do their work perfectly, and Lacomb records a death from double renal tuberculosis where no albumin had been present. Stress has been laid on the increased frequency of micturition by night as well as by day (Brown), but it is curious that nocturnal incontinence in children never seems to be due to this disease. Of late years the cystoscope has proved of the highest value in diagnosis, showing lesions in the bladder and about the mouths of the ureters, and enabling us to draw off the urine from one kidney separately. This latter aim can be attained also by a simpler instrument devised by Harris.[4] As to the hemorrhage, it appears sometimes at an early stage from congestion,[5] and later on from the erosions, and varies widely in amount. Unlike

[1] *Loc. cit.* [2] *Internat. M. Mag.*, 1896, iv. 881.
[3] *Centralbl. f. innere Med.*, 1896, xvii. 471.
[4] *J. Am. M. Ass.*, 1898, xxx. 236. [5] D. Newman, *Lancet*, 1898, ii. 14.

that caused by calculus, it is not increased by moving about. Sometimes by clots and sometimes from the *débris* of kidney tissue a ureter is blocked up, and thus we may find more or less complete retention alternating with profuse urination. This in turn may lead to hydronephrosis, but in other cases a tumour may be due to a collection of pus around the kidney, and often moderate enlargement of the organ takes place without suppuration.

If we turn to the question of the **bacilli**, there are two difficulties: first, their discovery at all, which may be impossible when there is much pus or blood present; and, secondly, their diagnosis from the smegma bacillus. Van Ketel[1] prefers to make a large number of specimens and stain twenty-four hours in an incubator with anilin water fuchsin, or if we can wait till the blood disappears the urine may be centrifugalised and stained rapidly with carbol fuchsin. If much pus is present, he shakes up with carbolic acid for five minutes, and allows it to settle, clearing the specimens by immersion in alcohol and ether. Von Jaksch recommends plate cultivations for separating the septic organisms present. For distinguishing the smegma bacillus, advantage is taken of the fact that it is decolourised by strong alcohol, while the tubercle bacillus is unaffected. Thus T. Brown, after Ziehl's fuchsin, employs a final bath of alcohol for more than five minutes; or we may counterstain with strong alcoholic methylene blue. Grünbaum finds the smegma bacillus in 50 per cent. of normal urines, and advises careful catheterisation to obtain urine free from it; but this seems hardly trustworthy, for the bacillus sometimes makes its home in the bladder.

With regard to **treatment**, pain is best relieved by codeia, according to Bryson,[2] who adds that cures are more often produced by antitubercular remedies than is generally believed. Among them he mentions sandal wood oil, cubebs, cod-liver oil, creasote, and especially change of climate. T. Brown has met with instances of surprising improvement even in advanced cases from rest, good food, change of climate, and large doses of creasote, and he claims that many early cases can be cured. Though, theoretically, it is desirable to get rid of a focus of disease in one kidney, yet the shock of operation may cause lesions elsewhere to spring into activity, and this point seems worthy to be taken into account. Savariaud[3] sums up the cases for operation as those where the patient is sinking from his sufferings or absorption of toxins, those where the kidney is practically destroyed by suppuration or where retention of urine or a peri-renal abscess exists, and finally some rare instances where hemorrhage or pain is so great that life is endangered. General miliary tuberculosis or advanced lung disease are a bar to operation, and all writers are agreed on the importance of avoiding catheterisation or washing out the bladder,

1 Quoted by Webb, *Brit. M. J.*, 1898, i. 1202. 2 *Loc. cit.*
3 *Gaz. d. Hôp.*, 1898, lxxi. 821.

which is sure to aggravate the trouble even when antiseptic precautions are taken. The bladder irritation is often not due to infection, but merely to the passage of *débris* from the kidney. Israel[1] considers that the condition of the second kidney is of more importance than that of the bladder with respect to operation, and if not tuberculous it may show amyloid changes or chronic nephritis. In such cases the diagnosis is most difficult.

The **mortality under surgical treatment** has been urged against it. Thus Facklam's statistics recorded a death-rate after nephrotomy of 60 per cent., and of 28 per cent. after nephrectomy. To discuss this subject would, however, be out of place here, but I may mention that Bolton Bangs,[2] after analysing 135 recent cases, speaks strongly in favour of surgical intervention. He claims that the immediate results are brilliant in relieving pain and prolonging life, and that the remote results are better than those of medical treatment. His figures show a death-rate of 20 per cent, or 29.6 per cent. if we include the nine months after operation. However, the fact is that no statistics of other methods exist and few of the many recoveries are reported. It would seem probable that with better diagnosis and improved hygienic methods a vastly greater success might be obtained without surgical aid.

Both in medical cases and when the fitness of a patient to undergo an operation has to be decided upon, the question often arises whether the kidneys are acting normally and excrete effete matters in due quantity. The symptoms of kidney disease are not always trustworthy, and a test has been devised which may have a certain value. This is the **injection of methylene blue**, which in health colours the water in half an hour, and shows increasing effects for three or four hours.[3] In kidney disease, except in acute epithelial nephritis, a delay in the occurrence of the tint is constantly found. Failure of a functional character is also met with where no lesions after death are demonstrable, and conversely, if the lesion is small and much healthy tissue remains, permeability may be normal. To carry out the test 1 c.c. of a one in twenty solution of pure methylene blue (not methyl blue) is injected into the gluteal muscles, and the bladder is emptied at the same time and again in half an hour, and then every hour afterwards. A colourless derivative is sometimes passed before or together with the blue. If urine containing this chromogen is heated with acetic acid a green tint appears. Achard and Castaigne believe that if both the chromogen and the blue are absent in the first specimens permeability is very feeble, but if the blue alone is delayed there is only a functional failure. The test seems to be quite harmless, and may throw light on the

[1] *Deutsche med. Wchnschr.*, 1898, xxiv. 443; abstract in *Post-Graduate*, 1898, xiii. 1066.

[2] *Ann. Surg.*, 1898, xxvii. 14.

[3] *Bull. et Mém. Soc. méd. d. Hôp. de Par.*, 1897, 3e sér., xiv. 637; abstract in *Med. Rec.*, 1897, lii. 554.

condition of many patients whose symptoms are obscure. Herter[1] thinks, however, that in advanced renal disease the injection is not without danger, and finds that in most individuals the colouration ceases in thirty-six hours. He adds that the disappearance of the dye in that time shows probably that the kidneys are normally clearing the blood of urea, salts, and other matters, although the urine may show albumin and casts. Numerous other papers have appeared on the subject.

The vexed question, What are the effete matters retained in **uræmia?** has had some light thrown on it by the experiments of Monari,[2] who finds that when excretion is defective there is not only an alteration in the density and corpuscles of the blood, but that the bactericidal power is lessened, and an important invasion of various microbes takes place. In 24 out of 30 cases he was able to find the B. coli, staphylococci, and other organisms in the blood, either alone or in combination. Thus uræmia is a septicæmia produced by various infections when the blood cells are weakened by the retention of any of the usual excreta, and the toxins thus formed are the causes of the various uræmic symptoms. Herter[3] points out that retention of potassium salts cannot be a cause of the toxicity of the serum in this state, for the serum contains very little of them. Nor is it due to urea or extractives, for the toxicity is lost at a temperature which has no effect on them. J. Rose Bradford, indeed, argues[4] that laboratory results do not settle the question, for experimenters can only by excision of the kidney produce "latent uræmia," such as is seen in suppression of urine either from obstruction or from paralysis of the kidney, and in this form nearly all the symptoms of true "acute uræmia" are absent. This absence may, however, be due to the fact that life is not prolonged sufficiently to produce the acute uræmia of Bright's disease. The retention theory, he agrees, is disproved by many facts, but he shows that there is a great disintegration of tissue in the muscles from altered metabolism and a formation of broken-up products in excess in the muscles. This, according to Monari, is due to the bacterial invasion which he has discovered, and it would seem that the toxins and not the urea and creatin are the active poisons.

Two practical points follow. One is the great **danger of massage** in gout and other quasi-uræmic states, because by it a large quantity of toxins may be suddenly driven out of the muscles into the circulation, producing what is known as "massage kidney"; and the other is the **value of bleeding** in uræmic asthma and allied conditions, if, as Herter says, the heart is strong, the tension high, and the anæmia not too profound. With it may be combined saline injections, at first

[1] *Phila. M. J.*, 1898, ii. 899.
[2] *Sperimentale*, 1897; abstract in *Brit. M. J.*, 1898, i. Epitome, p. 52.
[3] *Med. Rec.*, 1897, lii. 280. [4] *Lancet*, 1898, i. 919.

in moderate amount, but afterwards increased if the kidneys are able to react to them. Booth and Huchard[1] gave as much as 2,000 grammes daily with brilliant success in a case of surgical kidney, and with permanent relief. Copious enemata were also employed; but only moderate hypodermic injections of saline were used at first, till it was seen that the kidneys were sufficiently active. With anasarca and scanty urine, there is a danger that the fluid may fail to pass away and only increase the existing evils.

Von Noorden,[2] in speaking of the **diet** desirable **in chronic kidney disease**, points out that the distinction of white and dark meats rests on no scientific basis, and the restriction of patients to the former interferes uselessly with their appetite and comfort, while the physiologists find that the highest creatin values are in the white meat of chicken and rabbits. A more important statement is that patients with contracted kidneys and dilated hearts are remarkably relieved by restricting the fluid they take. Cardio-renal asthma is, he finds, extraordinarily relieved by this method, and the dilatation of the heart is lessened. He adds that in no stage of this disease is the elimination of the important products of tissue change lessened by the restriction of the fluids taken to 1¼ litres daily. He bases this statement on long-continued experiments, though he confesses it is opposed to the generally received view that flushing the kidney with water aids the patient.

GEORGE PARKER.

[1] *Bull. gen. de Thérap.* [etc.], 1897, cxxxii. 75.

[2] *Internat. M. Mag.*, 1899, viii. 325.

